# Upregulated lncRNA Cyclin‐dependent kinase inhibitor 2B antisense RNA 1 induces the proliferation and migration of colorectal cancer by miR-378b/CAPRIN2 axis

**DOI:** 10.1080/21655979.2021.1961656

**Published:** 2021-09-13

**Authors:** Yu Zheng, Jintao Zeng, Haoyun Xia, Xiangyu Wang, Hongyuan Chen, Liangxiang Huang, Changqing Zeng

**Affiliations:** a Department of Gastrointestinal Surgery, Fujian Provincial Hospital, Provincial Clinical Medical College of Fujian Medical University, Fuzhou, China; b Department of Clinical Medicine, School of Basic Medicine, Chengde Medical College, Chengde, China

**Keywords:** lncRNA CDKN2B-AS1, colorectal cancer, migration, proliferation, miR-378b, CAPRIN2

## Abstract

LncRNA Cyclin‐dependent kinase inhibitor 2B antisense RNA 1 (CDKN2B-AS1) plays a role in the progression of multiple cancers like cholangiocarcinoma, osteosarcoma and several gastrointestinal tumors. Few studies have linked its function and mechanism to the development of colorectal cancer (CRC). The expression of CDKN2B-AS1, microRNA (miR)-378b, and cytoplasmic activation/proliferation-associated protein 2 (CAPRIN2) was analyzed in CRC patients and cell lines. The proliferation and migration of CRC cells were evaluated after gain and loss-of function mutations. Interactions between CDKN2B-AS1 and miR-378b, miR-378b and CAPRIN2 were validated by luciferase reporter, RNA pull-down and RNA immunoprecipitation assays. The role of CDKN2B-AS1 was further confirmed in a xenograft mouse model. We found that the expression of CDKN2B-AS1 and CAPRIN2 was upregulated in CRC and they were linked to the poor differentiation and distant metastasis in CRC patients. CDKN2B-AS1 knockdown attenuated while CDKN2B-AS1 overexpression promoted CRC cell proliferation and migration. Notably, the results of Starbase 2.0 database analysis and in vitro experiments demonstrated that CDKN2B-AS1 could interact with miR-378b and regulate its expression. Furthermore, CAPRIN2 acted as a downstream target of CDKN2B-AS1/miR-378b that involved in modulating β-catenin expression in CRC cells. Upregulation of CDKN2B-AS1 contributed to CRC progression via regulating CAPRIN2 expression by binding to miR-378b. Downregulation of CDKN2B-AS1 suppressed tumor growth and Ki-67 staining in vivo that was related to the miR-378b/CAPRIN2 pathway. This study indicated that lncRNA CDKN2B-AS1 promoted the development of CRC through the miR-378b/CAPRIN2/β-catenin axis. CDKN2B-AS1 might serve as a potential and useful target in CRC diagnosis and treatment.

## 1. Introduction

Colorectal cancer (CRC) is a malignancy with high morbidity and mortality in the world, with about 1.4 million new cases and 0.7 million deaths in 2012 [1]. Researchers have estimated that the global burden of CRC will increase by 60% to more than 2.2 million new cases and 1.1 million cancer deaths yearly by 2030 [1]. Genetic mutations, dietary and lifestyle factors constitute the major causes for CRC development. Important advancements in CRC therapy have been achieved in recent years. Especially the exciting progress in immunotherapy provides more choices for advanced patients [2]. However, the survival rates of patients with distant metastasis have not been improved yet [3].

It has been estimated that about 75% of the human genome can transcribe into RNAs, but only 2% of them finally translate to proteins [4]. The functions of those non-coding RNAs have been extensively studied over the past decade. Long non-coding RNAs (lncRNAs) are one group of non-coding transcripts that contain more than 200 nucleotides. Based on their function, lncRNAs can be classified into signaling, decoy, guide, and scaffold lncRNAs, which regulate gene expression or protein activity via different mechanisms [5]. Signaling lncRNAs specifically influence cellular signaling pathways; decoy lncRNAs interact with and titrate away transcription factors from binding to the target gene promoters facilitating gene activation or silencing; guide lncRNAs direct different regulators to specific genomic loci for regulating gene expression; scaffold lncRNAs help to tether multiple proteins to form protein complexes [5]. At posttranslational levels, lncRNAs act as competitive endogenous RNAs and regulate the expression and activities of critical proteins in somatic cell reprogramming [6,7]. LncRNAs also contribute to cell malignant transformation, resulting in the initiation of multiple cancers, such as gastric cancer [8], lung cancer [9] and colorectal cancer [10]. Despite various lncRNAs are investigated, their functions in cancer progression still remain elusive.

Cyclin‐dependent kinase inhibitor 2B antisense RNA 1 (CDKN2B-AS1) or ANRIL is located at chromosome 9q21. It was initially reported to transcribe into a non-coding RNA that was involved in epigenetic modification of INK4b-ARF-INK4a genes [11]. Following that, the functions of CDKN2B-AS1 in many diseases were elucidated. A study demonstrated the association of CDKN2B-AS1 polymorphisms with the risk of coronary heart disease [12]. CDKN2B-AS1 might be an important regulator in diabetic nephropathy and diabetic cardiomyopathy [13]. CDKN2B-AS1 expression could be induced by high glucose [14]. It controlled the expression of extracellular matrix proteins and vascular endothelial growth factor in kidneys [13]. Most importantly, CDKN2B-AS1 might serve as a critical biomarker and target for cancer prognosis and treatment. CDKN2B-AS1 expression was enhanced in tumor tissues like laryngeal squamous cell cancer [15], ovarian cancer [16], hepatocellular carcinoma [17] and colorectal cancer [18]. Also, single nucleotide polymorphism in CDKN2B-AS1 gene was found to be associated with lung cancer risk [19]. This lncRNA could regulate cancer cell proliferation, metastasis, apoptosis, senescence or epithelial-mesenchymal transition through sponging downstream microRNAs (miRNAs) [20,21]. Sun et al. [18] found that CDKN2B-AS1 was overexpressed and acted as a promoter of the lymphangiogenesis and lymphatic metastasis in CRC. And its expression was closely associated with poor prognosis in CRC patients. Nevertheless, Hu and his coworkers revealed that CDKN2B-AS1 was down-regulated in human CRC tissues [18,22]. Besides, the effects of CDKN2B-AS1 on CRC cell proliferation and migration have not been well-understood.

This study aimed to explore the expression and function of CDKN2B-AS1 in CRC. Furthermore, the underling mechanism of CDKN2B-AS1 on the progression of CRC was investigated. Our data demonstrated that CDKN2B-AS1 could promote CRC cell proliferation and migration and facilitate CRC tumor growth through the miR-378b/cytoplasmic activation/proliferation-associated protein 2 (CAPRIN2) axis. These results provided evidence that CDKN2B-AS1 could be a useful target in CRC therapy.

## 2. Materials and methods

### 2.1. Human CRC and adjacent tissue samples

Human CRC and adjacent tissue samples were collected from 39 patients admitted in Fujian Provincial Hospital. All the patients were newly diagnosed with CRC and underwent surgical resection from June 1 to 30 December 2018. They did not receive chemotherapy or radiotherapy before the resection. Written informed consents were obtained from all the patients. This study was carried out with the approval of the Medical Ethics Committee of Fujian Provincial Hospital (No. 2018–015).

### 2.2. Cell culture

CRC cell lines like HT29, Caco2, SW480, and SW1116 as well as normal human colon epithelial cell line NCM460 were obtained from the American Type Culture Collection. CRC and epithelial cells were cultured with RPMI-1640 medium (Thermo Fisher Scientific, Shanghai, China) containing 10% fetal bovine serum (FBS; Thermo Fisher Scientific). Cells were cultured in a humid incubator with 5% CO_2_ at 37°C.

### 2.3. Vector construction and transfection

shRNAs targeting CDKN2B-AS1 (sh-CDKN2B-AS1), CAPRIN2 (sh-CAPRIN2) and their control shRNAs were designed and cloned into pGenesil-1 plasmid (LMAI Bio, Shanghai, China). To overexpress CDKN2B-AS1, the cDNA of CDKN2B-AS1 amplified from HT29 cells was cloned into pLV-EGFP(2A)Puro plasmid between XbaI and BamHI restriction sites.

miR-378b mimic, miR-378b inhibitor (anti-miR-378b) and their negative controls were synthesized by GenePharma (Shanghai, China). sh-CDKN2B-AS1, sh-CAPRIN2, miR-378b mimic, anti-miR-378b and their negative controls were transfected into CRC cells using Lipofectamine 2000 according to the manufactures’ instructions.

### 2.4. Cell proliferation assay

CRC cell viability was determined using Cell Counting Kit-8 (CCK-8) assay. Briefly, CRC cells (5 × 10^3^ cells/well) were placed into 24-well plates and incubated with 10% CCK-8 solution (MedChemExpress, Monmouth Junction, NJ, USA). The proliferation rate was determined at 24, 48, 72 and 96 h after transfection. The absorbance was measured at 450 nm using a microplate reader.

The proliferation of CRC cells was also determined by colony formation assay. CRC cells were seeded into 6-well plates (500 cells/well), gently rotated the plate to disperse the cells evenly. The cells were incubated at 37°C for 2 weeks. After fixing with paraformaldehyde for 10 min, the colonies were stained with crystal violet solution for 30 min at room temperature. Cell colonies were photographed by a light microscope and only colonies that included more than 50 cells were counted to analyze the clone formation rate.

### 2.5. Cell migration assay

CRC cell migration was evaluated using Transwell assay. Briefly, 1 × 10^4^ CRC cells transfected with sh-CDKN2B-AS1 or CDKN2B-AS1 overexpressing vector were plated into the upper surfaces of the Transwell chambers and incubated with serum-free RPMI 1640 medium. The medium with 20% FBS was added to the lower chamber. CRC cells were allowed to migrate for 24 h. The non-migrated cells were removed by a cotton swab. Whereas the migratory cells were fixed in 4% formaldehyde and stained with Giemsa. Cell numbers were counted under a microscope.

### 2.6. qRT-PCR analysis

The extraction of total RNA from CRC tissues and cell lines was performed using TRIzol reagent (Thermo Fisher Scientific) with the manufacturer’s protocol. RNA was reversely transcribed into cDNA using a reverse transcript kit (Takara, Beijing, China). qRT-PCR analysis was conducted with the TB Green Premix Ex Taq kit (Takara) using Applied Biosystems 7500 Fast Real Time PCR System (Thermo Fisher Scientific) following the manufacturer’s instruction. Gene expression was calculated using the 2^−ΔΔCt^ method, with normalization to U6 or glyceraldehyde-3-phosphate dehydrogenase (GAPDH). The primer sequences for qRT-PCR were listed in Table 1.

**Table 1.** The primer sequences for qRT-PCR

Gene symbol	Primer sequence
microRNA-378b	Reverse-transcribed primer: 5ʹ-GTC GTA TCC AGT GCA GGG TCC GAG GTA TTC GCA CTG GAT ACG AC T TCT GC-3ʹ Forward: 5ʹ-ACA CTC CAG CTG GGA CTG GAC TTG GAG-3ʹ reverse: 5ʹ-CGC AGG GTC CGA GGT ATT C-3’
miR-378e	Reverse-transcribed primer: 5ʹ-GTC GTA TCC AGT GCA GGG TCC GAG GTA TTC GCA CTG GAT ACG ACT CCT GA-3ʹ Forward: 5ʹ-ACA CTC CAG CTG GGA CTG GAC TTG GAG-3ʹ reverse: 5ʹ-CGC AGG GTC CGA GGT ATT C-3’
U6	Reverse-transcribed primer: 5ʹ-AAC GCT TCA CGA ATT TGC GT-3ʹ Forward: 5ʹ-CTC GCT TCG GCA GCA CA-3ʹ reverse: 5ʹ-AAC GCT TCA CGA ATT TGC GT-3’
CDKN2B-AS1	Forward: 5ʹ-GCG CCG GAC TAG GAC TAT TT-3ʹ reverse: 5ʹ-GCC AGG ACG GAG ATC AGA TG-3’
CAPRIN2	Forward: 5ʹ-TAA GGA TCG CCT GAA AAG TGG A-3ʹ reverse: 5ʹ-AGT TTT AGC ATG TGC TCC CTT C-3’
GAPDH	Forward: 5ʹ-GGA GCG AGA TCC CTC CAA AAT-3ʹ reverse: 5ʹ-GGC TGT TGT CAT ACT TCT CAT GG-3’

### 2.7. Western blot detection

To examine the expression of Ki-67, proliferating cell nuclear antigen (PCNA), CAPRIN2, and β-catenin, CRC cells were washed with PBS and lysed in Radioimmunoprecipitation Assay buffer (50 mM Tris pH 7.4, 150 mM NaCl, 1% NP-40, 0.5% deoxycholic acid, 0.1% SDS). Protein concentration was measured using a BCA protein assay kit (Beyotime, Shanghai, China). Approximately 40 μg of protein was separated by dodecyl sulfate, sodium salt (SDS)-Polyacrylamide gel electrophoresis and then electroblotted onto polyvinylidene fluoride membrane. To block nonspecific binding, the membrane was incubated in 5% nonfat milk for 1 h. Then, the membrane was incubated with primary antibodies including Ki-67 (1:1000), PCNA (1:1000), CAPRIN2 (1:1000), and β-catenin (1:1000) overnight at 4°C. All primary antibodies were provided by Abcam. After washing with TBS, the membrane was incubated with HRP-conjugated goat anti-mouse IgG (1:10,000) secondary antibody. The band density of protein was analyzed using ImageJ software (NIH, Bethesda, MD, USA), and GAPDH was used as the loading control.

### 2.8. Biotinylated RNA pull-down assay

Biotinylated RNA pull-down assay was used to validate the interaction between CDKN2B-AS1 and miR-378b, which was performed according to a previous study [23]. Briefly, CRC cells with CDKN2B-AS1 overexpression were transfected with biotin-miR-378b or biotin-miR-378b-Mut using Lipofectamine 2000 (Thermo Fisher Scientific). 48 h after transfection, CRC cells were harvested, lysed, and sonicated. The biotinylated RNA complex was pulled down by incubating the cell lysates with streptavidin C1 magnetic beads (Thermo Fisher Scientific) at 4°C overnight. After washing, the RNA complexes bound to the beads were eluted and extracted by TRIzol LS reagent (Thermo Fisher Scientific). The level of CDKN2B-AS1 that captured by biotin-miR-378b or biotin-miR-378b-Mut was quantified using qRT-PCR.

### 2.9. Dual‐luciferase reporter assay

Dual‐luciferase reporter assay was carried out as previously described [24]. The wild-type of CDKN2B-AS1 and CAPRIN2-3ʹUTR containing miR-378b binding sites as well as their mutant fragments were synthesized and inserted into pmirGLO luciferase reporter vector to generate pmirGLO-CDKN2B-AS1, pmirGLO-CDKN2B-AS1-Mut, pmirGLO-CAPRIN2-3ʹUTR, and pmirGLO-CAPRIN2-Mut reporter plasmids. SW480 cells were co-transfected with theses constructed reporter plasmids and miR-NC/miR-378b mimic using Lipofectamine 2000. 48 h after transfection, cells were harvested and dual-luciferase reporter assay system (Promega, WI, USA) was employed to determine the relative luciferase activity. Firefly luciferase activity was normalized to Renilla luciferase activity.

### 2.10. RNA immunoprecipitation (RIP) assay

The interaction between CDKN2B-AS1 and miR-378b was confirmed by RIP assay with Imprint RNA Immunoprecipitation Kit (Sigma-Aldrich, Shanghai, China) and Argonaute2 (AGO2) antibody (Abcam, Cambridge, UK) [17]. Briefly, SW480 cells were transfected with miR-378b mimic or control miRNA. 48 h after the transfection, cells were harvested and lysed with complete lysis buffer. Subsequently, cell lysates were incubated overnight at 4°C with magnetic beads conjugated with AGO2 antibody or IgG antibody, which was used as a negative control. After washing, immunoprecipitated CDKN2B-AS1 was purified and detected by qRT-PCR. The RIP fraction Ct value was normalized to the input RNA fraction Ct value.

### 2.11. Animal experiments

All animal experiments were approved by the Institutional Animal Care and Use Committee of Fujian Provincial Hospital (No. 2018–015). 4-week-old, specific-pathogen-free (SPF) male BALB/c nude mice were obtained from Shanghai SLAC Laboratory Animal CO. Ltd (Shanghai, China) and housed in the laboratory animal center of Fujian Medical University with free access to food and water. Mice were randomly divided into 2 group (n = 6 per group): the sh-Ctrl group and the sh-CDKN2B-AS1 group. SW480 cells (3 × 10^6^ cells/200 μL) stably transfected with sh-Ctrl or sh-CDKN2B-AS1 were subcutaneously injected into the BALB/c nude mice, respectively. Tumor volumes were calculated every 4 days by the following formula: tumor volume = width^2^ × length × 0.5. All mice were sacrificed 4 weeks after injection by cervical dislocation under isoflurane anesthesia. The xenografted tumors were removed for further analysis. A portion of tissues was stored at −80°C for the detection of CDKN2B-AS1, miR-378b, CAPRIN2 and β-catenin. And the other tumor tissues were embedded, fixed, and determined Ki-67 levels by immunohistochemistry assay as previously described [25].

### 2.12. Statistical analysis

All our data were expressed as mean ± SD and analyzed by SPSS17.0 software (IBM, Armonk, NY, USA) or GraphPad Prism 5.0 (GraphPad Software, San Diego, CA, USA). The differences between two groups were analyzed by the Student’s t-test, while those among three or more groups were analyzed by one-way analysis of variance. The relationship between CDKN2B-AS1 expression and clinical features was analyzed by Pearson’s Mann-Whitney U test or χ^2^ test. P value less than 0.05 was considered to be statistically significant.

## 3. Results

### 3.1. Upregulation of CDKN2B-AS1 in human colorectal cancers

**Figure 1.**
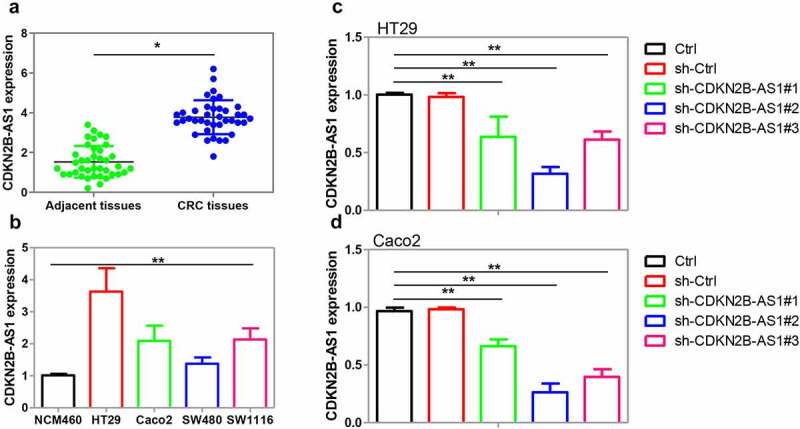
Examination of CDKN2B-AS1 expression in human CRC tissues and cell lines. (a) CDKN2B-AS1 expression was explored in human CRC tissues compared with adjacent normal tissues by qRT-PCR method, n = 39. (b) CDKN2B-AS1 expression was determined in CRC cell lines including HT29, Caco2, SW480, and SW1116 and a normal human colon epithelial cell line NCM460, n = 6. (c)(d) shRNAs targeting CDKN2B-AS1 were transfected into HT29 cells and Caco2 cells prior to the analysis of CDKN2B-AS1 expression, n = 6. *P < 0.05 compared with adjacent tissues, NCM460 cells or control cells. **P < 0.01 compared with NCM460 cells or control cells

Firstly, we detected CDKN2B-AS1 expression in human CRC tissues. From the results of qRT-PCR, we found that compared with their adjacent normal tissues (n = 39), CDKN2B-AS1 expression was increased in CRC tissues (n = 39) (Figure 1a). Besides, the associations between CDKN2B-AS1 expression and patients’ clinical characteristics were calculated. Our results indicated that CDKN2B-AS1 expression was high in cancer tissues with poor-differentiation and distant metastasis (Table 2). In addition, the expression of CDKN2B-AS1 was analyzed in CRC cell lines like HT29, Caco2, SW480, and SW1116 as well as in a normal human colon epithelial cell line NCM460. The figure showed that CDKN2B-AS1 expression was enhanced in HT29, Caco2, SW480, and SW1116 in comparison with that in NCM460 (Figure 1b). In all, our results revealed an upregulation of CDKN2B-AS1 expression in human CRC tissues and cell lines.

**Table 2.** Correlation between CDKN2B-AS1 and CRC patients’ clinical features

Characteristics	n (39)	CDKN2B-AS1 expression (%)		P value
		Low (n = 20)	High (n = 19)	
Gender				
Male	22 (56.4)	10 (50.0)	12 (63.2)	0.408
Female	17(43.6)	10 (50.0)	7 (36.8)	
Age				
<60	24 (61.5)	11 (55.0)	13 (68.4)	0.389
≥60	15 (38.5)	9 (45.0)	6 (31.6)	
Tumor Size				
<5 cm	18 (46.2)	11(55.0)	7 (36.8)	0.256
≥5 cm	21 (53.8)	9 (45.0)	12 (63.2)	
TNM stage				
I–II	15 (38.5)	10 (50.0)	5 (26.3)	0.129
III–IV	24 (61.5)	10 (50.0)	14 (73.7)	
Differentiation				
Low	15 (38.5)	5 (25.0)	10 (52.6)	0.045*
Moderate	13 (33.3)	6 (30.0)	7 (36.8)	
High	11 (28.2)	9 (45.0)	2 (10.6)	
Distant metastasis				
M0	14 (35.9)	12 (60.0)	2 (10.5)	0.001*
M1	25 (64.1)	8 (40.0)	17 (89.5)	

*P < 0.05.

### 3.2. CDKN2B-AS1 knockdown attenuates CRC cell proliferation and migration

**Figure 2.**
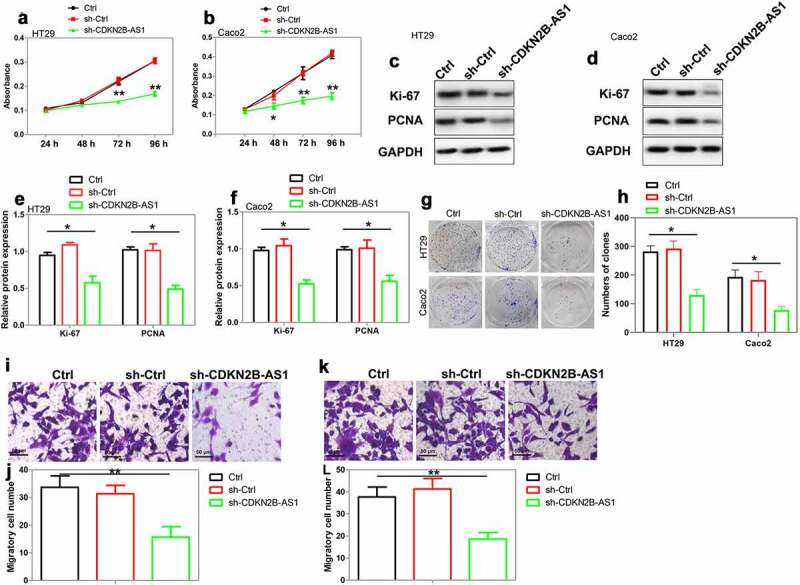
The effect of CDKN2B-AS1 knockdown on CRC cell proliferation and migration. (a)(b) Cell proliferation was evaluated by CCK-8 assay in HT29 cells and Caco2 cells after knockdown of CDKN2B-AS1, n = 6. (c)(d)(e)(f) Analysis and quantification of Ki-67 and PCNA protein expression by Western blot in HT29 cells and Caco2 cells after knockdown of CDKN2B-AS1, n = 4. (g)(h) Clone formation assay for the clone formation ability of HT29 and Caco2 cells after downregulation of CDKN2B-AS1, n = 4. (i)-(l) Detection of cell migration by Transwell assay in HT29 cells and Caco2 cells, n = 4. *P < 0.05, **P < 0.01 compared with control cells

Subsequently, we investigated whether CDKN2B-AS1 was involved in CRC cell proliferation and migration. Three shRNAs that designed to downregulate CDKN2B-AS1 were transfected into HT29 and Caco2 cells. The results of qRT-PCR showed that shRNAs transfection diminished CDKN2B-AS1 expression in these cells, of which shRNA#2 achieved the highest efficiency (Figure 1c,d). We then used shRNA#2 to suppress CDKN2B-AS1 expression in the following study. As indicated by CCK-8 assay, the proliferation of CRC cells was restrained by CDKN2B-AS1 shRNA but not by the control shRNA (Figure 2a,b). Ki-67 and PCNA are biomarkers for CRC cell proliferation and the poor prognosis of CRC patients [26]. The expression of Ki-67 and PCNA was suppressed after CDKN2B-AS1 shRNA transfection (Figure 2c–f). Clone formation assay revealed that knockdown of CDKN2B-AS1 significantly suppressed the number of HT29 and Caco2 clones when compared with the control (Figure 2g and h). The migration of CRC cells was also attenuated by CDKN2B-AS1 knockdown (Figure 2i–l). Taken together, these results indicated that CDKN2B-AS1 knockdown attenuated CRC cell proliferation and migration.

### 3.3. CDKN2B-AS1 overexpression promotes CRC cell proliferation and migration

**Figure 3.**
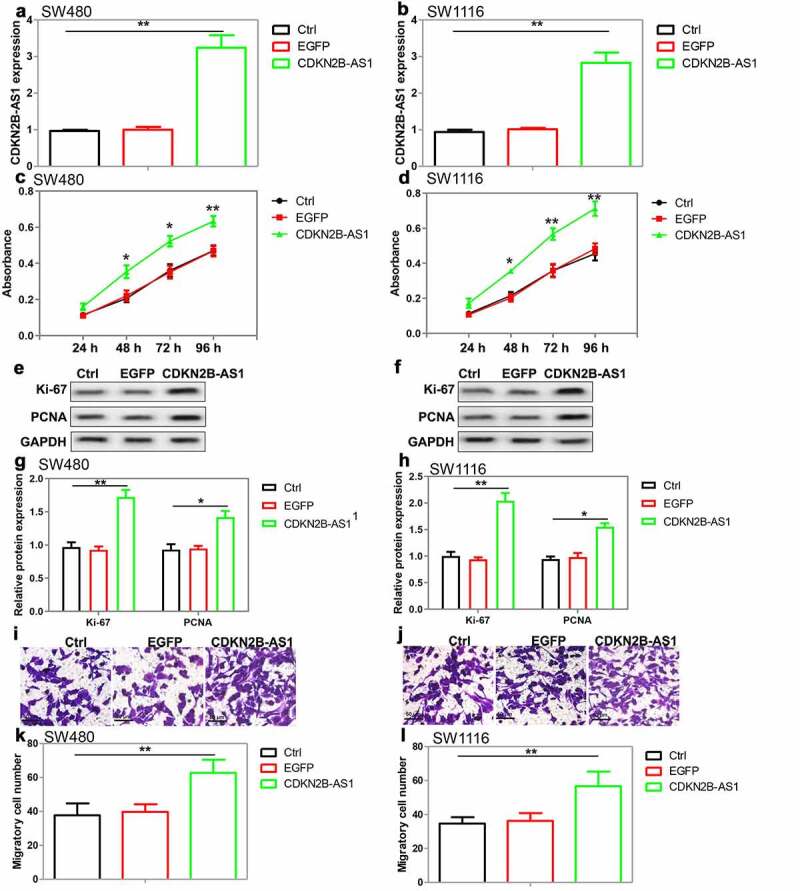
The effect of CDKN2B-AS1 overexpression on CRC cell proliferation and migration. (a)(b) Analysis of CDKN2B-AS1 expression in SW480 cells and SW1116 cells after lentivirus vectors transfection, n = 4. (c)(d) Evaluation of cell proliferation by CCK-8 assay in SW480 cells and SW1116 cells with CDKN2B-AS1 overexpression, n = 6. (e)(f)(g)(h) Analysis and quantification of Ki-67 and PCNA protein expression by Western blot in SW480 cells and SW1116 cells with CDKN2B-AS1 overexpression, n = 4. (i)(j) (k)(l) Transwell assay was used to detect the migration of SW480 cells and SW1116 cells with CDKN2B-AS1 overexpression, n = 4. *P < 0.05, **P < 0.01 compared with control cells

To further clarify the role of CDKN2B-AS1 in CRC cells, lentivirus vectors containing CDKN2B-AS1 were produced and then transfected into SW480 and SW1116 cells. The results of qRT-PCR showed that CDKN2B-AS1 expression was enhanced after the transfection (Figure 3a,b). CCK-8 assay showed that the proliferative ability was enhanced in CDKN2B-AS1-overexpressed SW480 and SW1116 cells compared with the control (Figure 3c,d). Meanwhile, CDKN2B-AS1 overexpression upregulated Ki-67 and PCNA expression in CRC cells (Figure 3e–h). Furthermore, transwell assay was conducted to explore cell migration and the results indicated that CDKN2B-AS1 overexpression accelerated the migration of CRC cells (Figure 3i–l). These data suggested that upregulation of CDKN2B-AS1 could promote the proliferation and migration of CRC cells.

### 3.4. CDKN2B-AS1 can interact with miR-378b in CRC cells

**Figure 4.**
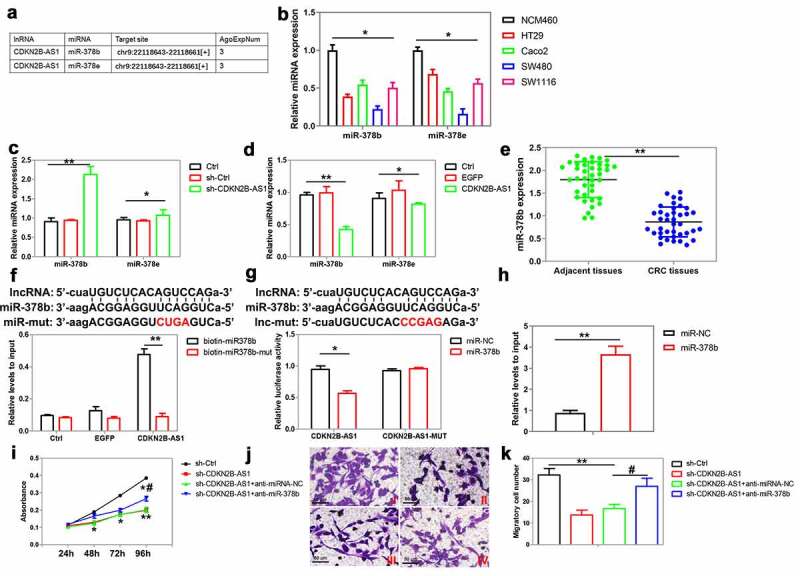
The interaction between CDKN2B-AS1 and miR-378b in CRC cells. (a) Starbase 2.0 database predicted that miR-378b and miR-378e were two microRNAs that potentially bind to CDKN2B-AS1. (b) Expression analysis of miR-378b and miR-378e in the normal human colon epithelial cell line NCM460 and colorectal cancer cells, n = 4. (c) (d) Analysis of miR-378b and miR-378e expression in SW480 cells with CDKN2B-AS1 knockdown and overexpression, n = 4. (e) The expression of miR-378b was analyzed in primary CRC tissues and adjacent tissues, n = 39. (f)The level of CDKN2B-AS1 captured by biotinylated wild-type/mutant miR-378b in CRC cells was analyzed using qRT-PCR, n = 3. (g) Interaction between CDKN2B-AS1 and miR-378b was validated by luciferase reporter assay, n = 4. (h) The enrichment of CDKN2B-AS1 with AGO2 was evaluated by RIP in CRC cells with miR-378b overexpression, n = 3. (i) CRC cell proliferation was detected after CDKN2B-AS1 knockdown with or without miR-378b inhibitor, n = 6. (j) (k) Observation of HT29 cell migration after CDKN2B-AS1 knockdown with or without miR-378b inhibitor, n = 4. I indicates control group, II indicates sh-CDKN2B-AS1 group, III indicates sh-CDKN2B-AS1+ anti-miRNA-NC group, IV indicates sh-CDKN2B-AS1+ anti-miR-378b group. *P < 0.05, **P < 0.01 compared with adjacent tissues or control cells, #P < 0.05 compared with sh-CDKN2B-AS1 group

Next, we explored the mechanism of CDKN2B-AS1 in regulating CRC cell proliferation and migration. With the help of Starbase 2.0 database (http://starbase.sysu.edu.cn/), we found that a series of miRNAs can bind to CDKN2B-AS1. Among them, miR-378b/miR-378e were two potential miRNAs that supported by at least 3 AGO CLIP-seq experiments, which help to identify miRNA targets with higher confidence (Figure 4a). The expression of miR-378b and miR-378e was obtained in CRC cells, including HT29, Caco2, Sw480 and SW1116. Their expression was relatively lower than the normal colon epithelial cells NCM460 (Figure 4b). SW480 cells were then selected in the following experiments, as the expression of miR-378b and miR-378e was the lowest in these cells (Figure 4b). In SW480 cells, CDKN2B-AS1 knockdown markedly promoted, and CDKN2B-AS1 overexpression reduced miR-378b expression; while the change of CDKN2B-AS1 expression moderately affected miR-378e expression (Figure 4c,d). Compared with adjacent normal tissues, a decrease of miR-378b expression was found in human CRC tissues (Figure 4e). Furthermore, miR-378b mimic and mutant miR-378b labeled with biotin at the 3ʹ end of their guide strands was transfected into SW480 cells, respectively. The results found that biotin-labeled miR-378b mimic but not mutant miR-378b captured more CDKN2B-AS1 from the lysates of CDKN2B-AS1-overexpressed CRC cells (Figure 4f). A dual luciferase report assay was used to further validate the interaction between CDKN2B-AS1 and miR-378b. The results showed that miR-378b mimic significantly decreased the luciferase activity in cells transfected with pmirGLO-CDKN2B-AS1 but not those with pmirGLO-CDKN2B-AS1-mut (Figure 4g). miRNAs exert their functions by associating with AGO2, which plays a critical role in the RNA induced silencing complex (RISC). We next confirmed the interaction between miR-378b and CDKN2B-AS1 via anti-AGO2 RIP. CDKN2B-AS1 was significantly enriched by anti-AGO2 antibody from CRC cells transfected with miR-378b mimic compared with the control miRNA (Figure 4h).

We further investigated whether CDKN2B-AS1 regulated CRC proliferation and migration through miR-378b. The results showed that the inhibition of proliferation and migration by CDKN2B-AS1 shRNA could be attenuated by miR-378b inhibitor in HT29 cells (Figure 4i–k). These results confirmed that CDKN2B-AS1 regulated CRC proliferation and migration through miR-378b.

### 3.5. Screening of downstream targets for miR-378b

**Figure 5.**
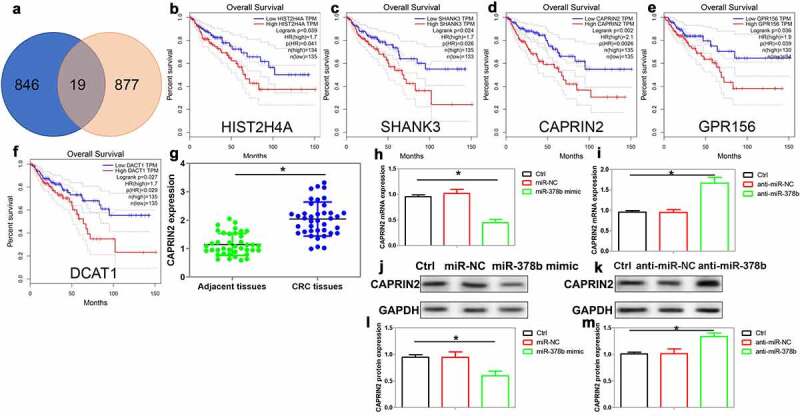
Screening of downstream targets for miR-378b in CRC cells. (a) Intersection between miR-378b potential target genes (Starbase 2.0) and genes associated with the overall survival of CRC patients (LinkedOmics database). (b)(c)(d)(e)(f) GEPIA database validated that HIST2H4A, SHANK3, CAPRIN2, GPR156, DCAT1 were significantly related to the overall survival of CRC patients. (g) Analysis of CAPRIN2 mRNA expression in CRC tissues and adjacent tissues, n = 39. (h) (j) (l) Exploration of CAPRIN2 mRNA and protein expression in SW480 cells after miR-378b mimic transfection, n = 4. (i)(k)(m) The mRNA and protein expression of CAPRIN2 was assayed in CRC cells after miR-378b inhibitor transfection, n = 4. *P < 0.05 compared with adjacent tissues or control cells

The target gene of miR-378b in CRC was further analyzed. To screen downstream targets for miR-378b, we obtained a list of candidate targets of miR-378b from starbase 2.0. The list contained 896 protein coding genes. We also acquired another gene list from the LinkedOmics database (http://www.linkedomics.org/). This database analyzed the association between mRNA expression and overall survival of patients with colorectal adenocarcinoma (TCGA_COADREAD) by Cox regression analysis. This list contained 865 protein coding genes that negatively affected patients’ overall survival. 19 intersected genes were found from these two gene lists (Figure 5a). The candidate genes included SOX7, SLC9A5, DCBLD2, HIST2H4A, SLC2A3, CLK2, PI4K2A, ZBTB20, NLGN2, NOL3, ZC3H6, AP2M1, PTPN14, SHANK3, INHBB, CAPRIN2, GPR156, POU2F1, and DACT1. Subsequently, the TCGA database using GEPIA software (http://gepia.cancer-pku.cn/) were employed to confirm their associations with the overall survival of colorectal adenocarcinoma patients. The results showed that patients with a higher expression of HIST2H4A, SHANK3, CAPRIN2, GPR156, and DACT1 possessed poor overall survival (Figure 5b–f).

**Figure 6.**
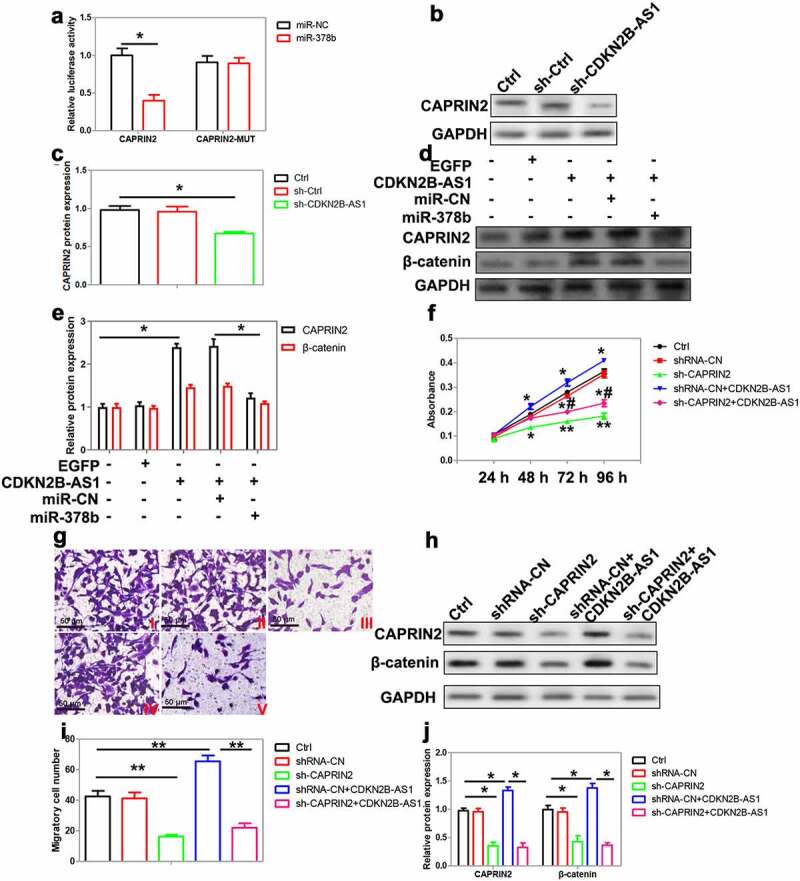
The role of CAPRIN2 in CRC cells. (a) The interaction between miR-378b and CAPRIN2 3ʹ-UTR was confirmed using the luciferase reporter assay, n = 4. (b)(c) The expression of CAPRIN2 was analyzed by Western blot in HT29 cells with CDKN2B-AS1 knockdown, n = 4. (d) (e)The levels of CAPRIN2 and β-catenin were measured by Western blot in SW480 cells after CDKN2B-AS1 overexpression with or without miR-378b, n = 4. (f) Delineating SW480 cell proliferation after CAPRIN2 knockdown with or without CDKN2B-AS1 overexpression, n = 6. (g)(i) SW480 cells were transfected with CAPRIN2 shRNA with or without CDKN2B-AS1 overexpression, and cell migration was observed by transwell assay, n = 4. I indicates control group, II indicates shRNA-NC group, III indicates sh-CAPRIN2, IV indicates shRNA-CN+CDKN2B-AS1 group, V indicates sh-CAPRIN2+ CDKN2B-AS1 group. (h)(j) Analysis of CAPRIN2 and β-catenin expression in SW480 cells after CAPRIN2 knockdown with or without CDKN2B-AS1 overexpression, n = 4. Except specially indicated, *P < 0.05, **P < 0.01 compared with the control group; #P < 0.05 compared with shRNA-CN+CDKN2B-AS1 group

Among these 5 selected targets, CAPRIN2 drew our attention as this protein has been reported to bind to low-density lipoprotein receptor-related protein 5 and 6 (LRP5/6) and promote Wnt signaling pathway transduction [27]. We found that CAPRIN2 expression was enhanced in CRC tissues compared with the control (Figure 5g). And the upregulation of CAPRIN2 was related to the advanced TNM stage, poor-differentiation and distant metastasis in CRC patients (Table 3). In CRC cells, miR-378b mimic decreased while miR-378b inhibitor enhanced CAPRIN2 expression (Figure 5h–m). A dual-luciferase reporter assay demonstrated that miR-378b mimic decreased the luciferase activity of pmirGLO-CAPRIN2 but not pmirGLO-CAPRIN2-Mut (Figure 6a). Knockdown of CDKN2B-AS1 significantly reduced CAPRIN2 protein expression in CRC cells (Figure 6b,c). Moreover, CDKN2B-AS1 overexpression enhanced while miR-378b mimic reduced CAPRIN2 expression in CRC cells (Figure 6d,e). The expression of β-catenin, an essential component of Wnt signaling pathway, was also increased by CDKN2B-AS1 and decreased by miR-378b mimic (Figure 6d,e). These results indicated that CAPRIN2 was a target of miR-378b in CRC.

**Table 3.** Correlation between CAPRIN2 and CRC patients’ clinical features

Characteristics	n (39)	CAPRIN2 expression (%)		P value
		Low (n = 20)	High (n = 19)	
Gender				
Male	22 (56.4)	13 (65.0)	9 (47.4)	0.267
Female	17(43.6)	7 (35.0)	10 (52.6)	
Age				
<60	24 (61.5)	11 (55.0)	13 (68.4)	0.389
≥60	15 (38.5)	9 (45.0)	6 (31.6)	
Tumor Size				
<5 cm	18 (46.2)	12(60.0)	6 (31.6)	0.075
≥5 cm	21 (53.8)	8 (40.0)	13 (68.4)	
TNM stage				
I–II	15 (38.5)	12 (60.0)	3 (15.8)	0.005*
III–IV	24 (61.5)	8 (40.0)	16 (84.2)	
Differentiation				
Low	15 (38.5)	3 (15.0)	12 (63.2)	0.008*
Moderate	13 (33.3)	9 (45.0)	4 (21.0)	
High	11 (28.2)	8 (40.0)	3 (15.8)	
Distant metastasis				
M0	14 (35.9)	11 (55.0)	3 (15.8)	0.011*
M1	25 (64.1)	9 (45.0)	16 (84.2)	

*P < 0.05.

### 3.6. CDKN2B-AS1 modulates CRC cell proliferation and migration through CAPRIN2

To examine the role of CAPRIN2 in CDKN2B-AS1-regulated CRC cell proliferation and migration, CAPRIN2 shRNA was transfected into SW480 cells with or without CDKN2B-AS1 overexpression. CAPRIN2 knockdown significantly inhibited cell proliferation and migration (Figure 6f, g,i). Downregulation of CAPRIN2 also downregulated β-catenin expression in SW480 cells (Figure 6h,j). Interestingly, the promotion of cell proliferation and migration as well as the augment of CAPRIN2 and β-catenin expression by CDKN2B-AS1 could be reversed by CAPRIN2 silencing (figure 6f–j). Thus, CDKN2B-AS1 might regulate CRC cell proliferation and migration through upregulating CAPRIN2.

### 3.7 Downregulation of CDKN2B-AS1 suppresses tumor growth in a xenograft mouse model

**Figure 7.**
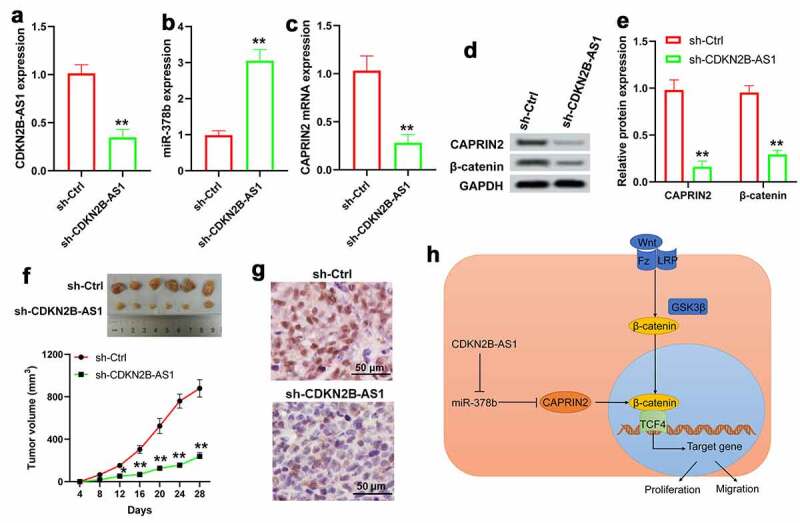
Downregulation of CDKN2B-AS1 suppressed tumor growth in a xenograft mouse model. qRT-PCR assay for the expression of CDKN2B-AS1 (a), miR-378b (b) and CAPRIN2 (c). (d) and (e) Western blot was used to detect the protein levels of CAPRIN2 and β-catenin in tumors. (f) Tumor images of mice after sacrificed as well as tumor volume. (g) Immunohistochemical staining for Ki-67. (h) Possible mechanism of CDKN2B-AS1 in CRC through miR-378b mediated regulation of CAPRIN2 and β-catenin expression. n = 6. *P < 0.05, **P < 0.01 compared with the sh-Ctrl group

The effect of CDKN2B-AS1 on CRC was further confirmed in a xenograft mouse model. We found that CDKN2B-AS1 shRNA notably decreased CDKN2B-AS1 expression in tumor tissues, accompanied by an increase of miR-378b and a decrease of CAPRIN2 (Figure 7a–e). Downregulation of CDKN2B-AS1 significantly reduced tumor volume and Ki-67 staining (Figure 7f and g). Additionally, the expression of β-catenin was inhibited in mice after CDKN2B-AS1 knockdown (Figure 7d and e). These results indicated that downregulation of CDKN2B-AS1 could inhibit CRC tumor growth that was related to the miR-378b/CAPRIN2/β-catenin pathway.

## 4. Discussion

LncRNAs play major roles in regulating cellular functions. The diagnostic and prognostic values of lncRNAs have been studied in some clinical trials recently [28]. However, much more data should be collected to establish their applicability toward clinical using. LncRNA CDKN2B-AS1 is widely expressed in many normal human tissues [17,29,30]. Importantly, the dysregulation of CDKN2B-AS1 has been linked to a variety of human cancers [17,30]. Our study sought to explore the expression and function of CDKN2B-AS1 in human CRC. We found that CDKN2B-AS1 was increased in CRC tissues and cell lines. CDKN2B-AS1 expression might be linked to the poor differentiation and distant metastasis of CRC. CDKN2B-AS1 knockdown suppressed, while CDKN2B-AS1 overexpression promoted CRC cell proliferation and migration. We also found that silencing of CDKN2B-AS1 suppressed tumor growth in a xenograft mouse model.

CDKN2B-AS1, like other lncRNAs, may act as miRNA sponges in CRC cells [30,31]. Here, analysis in Starbase 2.0 showed that CDKN2B-AS1 might interact with miR-378b and miR-378e. miR-378b and miR-378e are two members belonging to miR-378 family. miR-378b regulated the proliferation, migration and differentiation of keratinocytes [32]. Furthermore, the involvement of miR-378 in multiple tumors including cervical cancer [33], lung adenocarcinoma [34], and gastric cancer [35] has been elucidated in many literatures. Downregulation of miR-378 in CRC tissues has been reported [36]. The expression of miR-378 was inversely correlated with clinical stages of CRC patients and it might be a potential diagnostic and prognostic marker [37]. miR-378 overexpression could control CRC cell proliferation and invasion both in vitro and in vivo [38]. Interestingly, our data showed that CDKN2B-AS1 knockdown or overexpression markedly affected miR-378b expression but moderately regulated miR-378e expression. This might suggest that miR-378b was the miRNA binding to CDKN2B-AS1. The interaction between CDKN2B-AS1 and miR-378b was further confirmed by dual-luciferase reporter, RNA pull-down and RIP assays. Importantly, the inhibition of cell proliferation and migration by CDKN2B-AS1 knockdown could be reversed by miR-378b suppression. Additionally, downregulation of CDKN2B-AS1 caused the upregulation of miR-378b expression in mouse tumors. The results revealed that the impact of CDKN2B-AS1 on CRC was, at least partially, dependent on miR-378b.

Our data showed that miR-378b could directly bind to CAPRIN2 and affect its expression in CRC cells. CDKN2B-AS1 overexpression induced while miR-378b mimic inhibited CAPRIN2 expression. CAPRIN2 is a protein belonging to the C1q and tumor necrosis factor super-family of proteins. CAPRIN2 could be induced by fibroblast growth factor during lens fiber cell differentiation [39]. Conditional deletion of CAPRIN2 expression in mouse resulted in notable ocular defects [40]. C1q-related domain of CAPRIN2 could form a homotrimer via binding to calcium. This structure was essential for CAPRIN2 in regulating the canonical Wnt signaling [27,41]. CAPRIN2 R968/S969C, a gain-of-function mutation, actively regulated nuclear accumulation of β-catenin and promoted hepatoblastoma cell proliferation [42]. In the present study, we found that CAPRIN2 was increased in CRC tissues, and higher CAPRIN2 was associated with the advanced TNM stage, poor-differentiation and distant metastasis in CRC patients. Downregulation of CAPRIN2 reduced the proliferation and migration of CRC cells, which suggested that CAPRIN2 was an oncogene in CRC. Our results also found that CAPRIN2 knockdown in CRC cells suppressed β-catenin accumulation induced by CDKN2B-AS1, along with the inhibition of cell proliferation and migration. Moreover, downregulation of CDKN2B-AS1 led to the suppressed expression of CAPRIN2 and β-catenin in mouse tumors. The results seemed to be in line with a previous study [42] and demonstrated a regulation role of CAPRIN2 in β-catenin expression. Although it was not shown here, β-catenin would form a complex with TCF-4 and direct downstream genes to promote CRC cell growth, survival and invasion [43,44].

## 5. Conclusions

Our study indicated that lncRNA CDKN2B-AS1 was upregulated in human CRC and it promoted the progression of CRC through the miR-378b/CAPRIN2/β-catenin pathway (Figure 7h). Given that genetic heterogeneity is common in human CRC, the clinical significance of CDKN2B-AS1 needs to be verified in larger groups of patients, which may be from other clinical centers.

## Highlights

- CDKN2B-AS1 was upregulated of in human CRC.
- CDKN2B-AS1 promoted CRC cell proliferation and migration as well as tumor growth.
- CDKN2B-AS1 acted as a sponge of miR-378b in CRC.
- CAPRIN2 was a target of miR-378b in CRC.
- CDKN2B-AS1 functioned via the miR-378b/CAPRIN2/β-catenin pathway.
